# Effectiveness of long-term opioid therapy among chronic non-cancer pain patients attending multidisciplinary pain treatment clinics: A Quebec Pain Registry study

**DOI:** 10.1080/24740527.2018.1451252

**Published:** 2018-04-19

**Authors:** Hichem Saïdi, M. Gabrielle Pagé, Aline Boulanger, Mark A. Ware, Manon Choinière

**Affiliations:** a Centre de recherche du Centre hospitalier de l’Université de Montréal (CRCHUM), Montreal, Quebec, Canada; b Department of Pharmacology, Faculty of Medicine, Université de Montréal, Montreal, Quebec, Canada; c Department of Biomedical Sciences, Faculty of Medicine, Université de Montréal, Montreal, Quebec, Canada; d Department of Anesthesiology, Faculty of Medicine, Université de Montréal, Montreal, Quebec, Canada; e Centre d’expertise en gestion de la douleur du Réseau universitaire intégré en santé de l’Université de Montréal, Montreal, Quebec, Canada; f Québec Pain Research Network; g Alan Edwards Center for Research on Pain, McGill University, Montreal, Quebec, Canada; h Department of Family Medicine, Faculty of Medicine, McGill University, Montreal, Quebec, Canada; i Department of Anesthesia, Faculty of Medicine, McGill University, Montreal, Quebec, Canada

**Keywords:** opioids, chronic pain, Quebec Pain Registry, multidisciplinary pain treatment facility, treatment effectiveness

## Abstract

**Objective:**

The objective of this study was to investigate in a real-life context the effectiveness of long-term opioid therapy for reducing pain intensity and interference and improving health-related quality of life (QOL) in patients with chronic noncancer pain.

**Methods:**

Participants were 893 patients (age = 52.4 ± 14.1, female = 62.4%) enrolled in the Quebec Pain Registry (2008–2011) who completed questionnaires before their first visit at one of three multidisciplinary pain management clinics and 6 and 12 months thereafter. Based on their opioid use profile (OUP), patients were categorized as nonusers, non-lasting users, or lasting users. Data were analyzed using generalized estimating equations.

**Results:**

More than 60% of patients newly initiated on opioid therapy stopped their medication mainly because of adverse effects and/or lack of pain relief. OUP significantly predicted pain intensity and interference and physical QOL (pQOL; *P* values < 0.001). Lasting users of opioids reported higher levels of pain intensity and interference and poorer pQOL than nonusers and/or non-lasting users over the 12-month follow-up (*P* values < 0.001). However, all effect sizes were small, thus questioning the clinical significance of these group differences. Among lasting users, more than 20% of patients experienced a meaningful amelioration in pain intensity and interference as well as mental QOL (mQOL), whereas only 8% exhibited improved pQOL.

**Discussion:**

A significant subgroup of patients may benefit from long-term opioid therapy in terms of pain severity and mQOL but the majority do not. The challenge facing clinicians is how to identify who the responders will be.

## RÉSUMÉ

**Objectif**: L'objectif de cette étude était d'investiguer dans un contexte de vraie vie l'efficacité à long terme des opioïdes pour réduire l'intensité et l'interférence de la douleur et améliorer la qualité de vie reliée à la santé (QDV) des patients souffrant de douleur chronique non cancéreuse.

**Méthodes**: Les participants à cette étude étaient 893 patients (âge = 52.4 ± 14.1, femmes = 62,4%) enrôlés dans le Registre Québec Douleur (2008–2011) et qui avaient complété des questionnaires avant leur premiére visite dans l'un des trois centres multidisciplinaires de gestion de la douleur ainsi qu’à 6 et 12 mois plus tard. Selon leur profil d'utilisation d'opioïdes (PUO), les patients ont été classés en non-utilisateurs, utilisateurs non continus, ou utilisateurs continus. Les données ont été analysées à l'aide de modéles d'équation d'estimation généralisée.

**Résultats**: Plus de 60% des patients nouvellement initiés à une thérapie à base d’opioïdes en avaient cessé la prise principalement à cause des effets indésirables et/ou d’un manque de soulagement de leur douleur. Le PUO prédisait d’une maniére significative l'intensité de la douleur et son interférence ainsi que la QDV physique (QDVp; valeurs p < 0.001). Comparés aux non-utilisateurs et/ou utilisateurs non continus, les utilisateurs continus rapportaient des niveaux plus élevés d'intensité et d’interférence de la douleur ainsi qu’une moins bonne QDVp au cours des 12 mois de suivi (valeurs p < 0.001). Cependant, la magnitude de ces effets était de petite taille, remettant ainsi en question la signification clinique des différences observées entre ces groupes. Parmi les utilisateurs continus, plus de 20% d'entre eux montraient une amélioration significative de leur condition douloureuse en termes d'intensité et d’interférence ainsi que de la QDV mentale (QDVm), alors que seulement 8% présentaient une amélioration de leur QDVp.

**Discussion**: Un sous-groupe important de patients peut bénéficier d’un traitement à long terme à base d’opioïdes en termes de sévérité de la douleur et de QDVm, mais ce n’est pas le cas pour la majorité des patients. Le défi auquel les cliniciens doivent faire face est de déterminer les patients les plus susceptibles de bénéficier de ce type de traitement.

## Introduction

In Canada and the United states, opioids (e.g., morphine, oxycodone, fentanyl) are among the most widely used drugs to treat chronic noncancer pain (CNCP) along with acetaminophen and nonsteroidal anti-inflammatory drugs.^1–3^ Opioids are potent analgesics, but their use is associated with several side effects such as respiratory depression, nausea, or constipation.^4^ Over the past 2 decades and until 2010, the use of opioids for CNCP has dramatically increased,^5,6^ as have associated serious damages such as overdose, abuse, or addiction.^7–12^ However, since 2011, the general use of opioids and rates of death related to their prescription tend to decrease,^6^ whereas the death rates due to illicit use of opioids have increased.^13^

There is a lack of evidence in the literature supporting the effectiveness of long-term opioid therapy among CNCP patients. A literature review published in 2009 by the American Pain Society in collaboration with the American Academy of Pain Medicine found that very few studies have investigated the long-term benefits of opioids (≥6 months) in CNCP,^14^ among which two high-quality systematic reviews found that opioids were discontinued by a high proportion of patients due to adverse events or insufficient pain relief.^15,16^ Noble et al. concluded that only weak evidence supports the fact that patients who are able to continue opioids on a long-term basis experience clinically significant pain relief, though the evidence for improvement in health-related quality of life and physical functioning is inconclusive.^16^ The most recent literature review published in 2015 came to similar conclusions.^12^ To date, research on pharmacological agents for chronic pain management has been limited mainly to clinical trials, which are often of short duration and have stringent selection criteria.^17–19^ Furlan et al. reported that 74% of randomized clinical trials on opioid therapy had a duration of less than 6 weeks.^20^ Some cross-sectional studies have evaluated the effectiveness of long-term opioid therapy for improving pain severity and/or quality of life among patients with CNCP. Two studies found that patients with CNCP using long-term opioid therapy reported more pain and greater disability than those not using opioids.^21,22^ Two other studies, conducted among long-term opioid users only, found that patients reported relatively high pain intensity and interference,^23,24^ with an important proportion of patients having a deteriorated quality of life.^23^

Recently, Moulin et al. conducted a long-term national study in patients suffering from neuropathic pain and found that those who were on high dose of opioids at baseline and at follow-up exhibited poorer outcomes at 12-month follow-up.^25^ These authors therefore concluded that opioid therapy may not be beneficial in the majority of patients with chronic neuropathic pain.^25^ A subsequent subanalysis of this study, conducted by Bostick et al. involving 537 patients with chronic neuropathic pain, found that physical functioning and disability did not improve in patients who were prescribed opioids compared with those who were not.^26^ In contrast, Watson et al. surveyed 84 patients using opioids on a regular basis and found that the majority reported at least 50% or greater pain relief and a moderate improvement in disability.^27^ However, this study involved highly selected patients and the authors acknowledge that their results may not be generalizable to all CNCP patients in whom opioids are being initiated. Brooks et al. conducted a qualitative study focusing on the lived experience of nine patients using opioids to manage their CNCP came to the conclusion that positive effects of opioids outweighed the negative for most participants.^28^ However, further observational studies involving large samples of patients suffering from a variety of CNCP syndromes are needed in this field as the results can complement those of randomized clinical trials for evidence-based guidance of treatment decisions.

The objective of the present longitudinal study was to investigate in a real-life context the effectiveness of long-term opioid therapy for reducing pain severity (intensity and interference) and improving health-related quality of life (physical and mental) in patients with CNCP over a 1-year period.

## Materials and methods

### Participants

Participants were recruited from the Quebec Pain Registry (QPR), which is a vast database of patients referred to university-affiliated multidisciplinary pain treatment clinics in the province of Quebec, Canada.^29^ Patients were enrolled in the QPR between October 2008 and November 2014 if they were (1) scheduled for a first visit at the pain clinic for multidisciplinary treatment considerations, (2) aged 18 years or more, (3) fluent in spoken and written French and/or English, and (4) physically and cognitively able to complete questionnaires. Biopsychosocial data were collected prior to the initial visit at the pain clinic (baseline) and 6 months later. Up to March 2012, additional follow-up data were gathered at 12 and 24 months but only in those patients who had not been discharged from the pain clinic in the meantime, mainly because they do not have a family physician. Patients suffering from CNCP (≥3 months) were therefore selected for the present study among those enrolled in the QPR between October 2008 and April 2011. Only patients who provided written consent for their QPR data to be used for research purposes (more than 90% of patients^29^) and who were not currently taking opioids at the time of their first visit at the pain clinic were included in the study. Given small sample size at 24-month follow-up due to patients’ discharge in the meantime, only data collected up to 12 months were taken into account in the present study.

### Procedure

The Research Ethics Boards of the Centre hospitalier de l’Université de Montréal, McGill University Health Center, and Centre hospitalier de l’Université de Sherbrooke approved the QPR project. Data at baseline and follow-up (e.g., pain intensity and interference, health-related quality of life) were collected with a self-report questionnaire (patient self-administered questionnaire) and medical/clinical data (e.g., pain duration, pain diagnosis, analgesic medication, etc.) were gathered by the QPR nurses using a structured interview protocol (nurse-administered questionnaire).

### Questionnaires

All of the questionnaires were structured so that patients who reported multiple pain sites were asked to focus on the most painful one to complete the questionnaires.

#### Patient self-report questionnaires

##### Numeric Rating Scale for pain intensity

The Numeric Rating Scale (NRS)^30^ is a widely used scale to measure pain intensity.^31^ It consists of an 11-point scale (0 = *no pain*; 10 = *worst possible pain*) on which the participants are asked to select the number corresponding to the intensity of their pain. The NRS has good reliability, validity, and sensitivity.^30,31^ Participants were asked to rate on the NRS their average and worst pain intensity over the past 7 days at each time point (baseline and follow-ups).

##### Pain interference items of the Brief Pain Inventory

The Brief Pain Inventory (BPI-10)^32^ consists of ten items (as opposed to seven items in the original version of BPI^33,34^) assessing the extent to which pain impacts on various aspects of daily living. Participants are asked to rate on a scale from 0 (*does not interfere*) to 10 (*completely interferes*) the extent to which pain has interfered in the past 7 days with general activity, mood, mobility, normal work, relationships with others, sleep, enjoyment of life, self-care, recreational activities, and social activities.^32^ A global interference score can be derived by adding the ratings on all items, with higher scores indicating greater pain interference. The psychometric qualities of the BPI are well documented^32,33^ and it has been shown to have good validity and sensitivity to change in chronic pain patients attending a multidisciplinary pain treatment clinic.^35^ The BPI has been translated into French using a forward–backward translation method.^36^

##### Short-Form-12 Health Survey Version 2

The Short-Form-12 Health Survey Version 2 (SF-12v2)^37^ is a valid and reliable 12-item scale that assesses health-related quality of life (QOL). For each item, patients are asked to choose the answer that best describes their condition. The SF-12v2 generates norm-based scores for eight different domains as well as two composite scores representing physical and mental health–related QOL. Higher scores indicate better QOL.

#### Nurse-administered questionnaires

##### Pain history information and medication

Patients were asked information on their pain history (e.g., pain duration and frequency) and type(s) of medication currently used and used in the past 6 months to treat their pain.

##### Douleur Neuropathique 4

The Douleur Neuropathique 4 (DN4)^38^ is a well-validated screening tool that assesses the presence of neuropathic pain through self-report and physical examination. Each of the ten items is answered yes (score 1) or no (score 0). The total score is calculated as the sum of the ten items and the cutoff value for the presence of neuropathic pain is a total score of four out of ten.

##### Pain diagnosis

A summary of the data collected with the patient and nurse questionnaires was given to the pain physician at the patient’s initial visit. Once the visit was over, the physician was provided with a standardized form on which she or he was invited to record the patient’s diagnostic code(s) using the QPR pain diagnostic grid.^39^

### Opioid use profile and type of pain classification

The opioid use profile (OUP) variable was created by classifying patients into one of three categories based on current and past medication reported at each time point (baseline, 6- and 12-month follow-up). All patients included in the study were opioid naïve at baseline. Patients who reported not taking any opioids during the course of the study (at baseline, 6 and 12 months) were categorized as nonusers. Patients who started using opioids within the first 6 months but stopped taking them thereafter (at 6 or 12 month) were categorized as non-lasting users. Patients who were put on opioids within the first 6 months and continued taking them at each follow-up period (6 and 12 months) were categorized as lasting users.

A type of pain variable was also created and was composed of the three following categories: patients who were diagnosed with neuropathic pain by the physician of the pain clinic and had a DN4 score ≥ 4 were classified into the neuropathic pain category. Patients with a neuropathic pain diagnosis and DN4 score < 4 as well as those with a nonneuropathic pain diagnosis and DN4 score ≥ 4 were categorized as having mixed evidence of neuropathic pain. Last, patients with a pain diagnosis other than a neuropathic origin and a DN4 score < 4 were categorized as having nonneuropathic pain.

### Data analysis

Independent Student’s *t* tests and Pearson’s chi-square tests were employed to compare the baseline characteristics of patients who did and did not complete all questionnaires at each time point (baseline, 6 and 12 months). Mean and standard deviations along with frequency tables were used to describe participants’ characteristics. All patients included in this study were not using opioids at baseline.

Generalized estimating equations (GEEs) adjusting for sex, age, pain duration, and frequency were used to examine whether OUP and type of pain were associated over the 1-year follow-up period (time effect) with reported average and worst pain intensities, pain interference global scores, physical health–related quality of life (pQOL), and mental health–related quality of life (mQOL). In our five GEE models, the time effect encompassed measures collected at baseline, 6 months, and 12 months, thereby allowing group comparisons over time and at each time point in the event of a significant interaction. Pairwise comparisons were then used to assess the statistical significance of the group differences.

Effect sizes of group differences (Cohen’s *d*^40^ for continuous variables, the phi (φ) statistic for binary categorical variables, and Cramér’s *v* for discrete variables with more than two categories^41^) were also examined given that significant testing in studies involving large sample sizes like the present one can be misleading because even small differences can reach statistical significance, whereas clinically, they can be viewed as trivial and not meaningful.^42–44^ Only differences reaching a Cohen’s *d* value equal to or greater than  ±0.5 or a φ or a Cramér’s *v* value equal to or greater than  ±0.3 were considered meaningful and clinically important.^42–44^

Frequency tables were used to determine the proportion of lasting opioid users whose pain condition and quality of life improved, remained stable, or deteriorated. Based on the IMMPACT recommendations, a change of 20% or more in pain intensity and interference was considered as meaningful.^45^ For SF-12v2 quality of life scores, we considered a change of at least one SD of the mean norm-based scores of the general population as a meaningful change.

## Results

**Figure 1.**
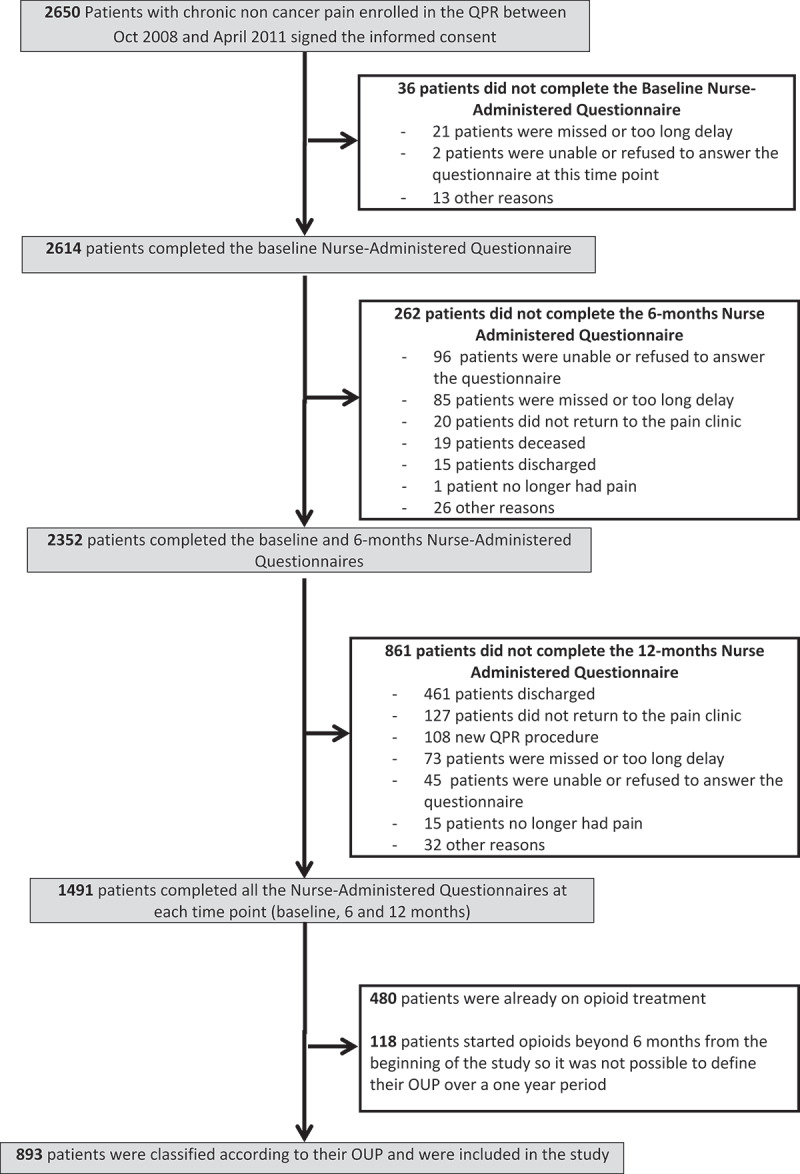
Study flow diagram.

A total of 2650 patients were enrolled in the QPR during the selected study period and consented for their information to be used for research purposes. As shown in Figure 1, a total of 893 opioid-naïve patients completed all of the nurse-administered questionnaires at each time point (baseline, 6 months, 12 months), so it was possible to categorize them according to their OUP. More than half of them (51.5%) were classified as non-opioid users, 30.6% as non-lasting users, and 17.9% as lasting users. Results of the statistical analysis comparing participants who did and did not complete all of the questionnaires at each time point (baseline, 6 and 12 months) in terms of age, sex, pain duration, type of pain, average and worst pain intensity, pain interference, pQOL, or mQOL at baseline revealed some statistically significant differences, but all of the effect sizes were small (Cohen’s *d *< 0.5; φ or Cramér’s *v* value *<* 0.3), suggesting that these differences were not clinically meaningful.

### Descriptive statistics

Table 1 shows the demographic and pain characteristics of the entire sample of participants and for each patient’s OUP category. Participants’ ages ranged from 18 and 92 years with a median of 51 years, and 62% were female (*n* = 554). Patients suffered from CNCP from 3 months to 60 years, the median being 3 years. More than 75% of the participants were classified as having neuropathic pain or mixed evidence of neuropathic pain. At baseline, pain intensity on the average in the past 7 days was 6.75 (SD = 1.9) on the 0–10 NRS.

**Table 1.** Patient demographics and pain characteristics at baseline according to the opioid use profile and for the total sample.

	Nonusers	Non-lasting users	Lasting users	Total
	*N* (%)	*N* (%)	*N* (%)	*N* (%)
*N*	460 (51,5%)	273 (30,6%)	160 (17,9%)	893
Sex				
Females	284 (61,7%)	176 (64,5%)	94 (58,8%)	554 (62%)
Males	176 (38,3%)	97 (35,5%)	66 (41,2%)	339 (38%)
Age				
Age≤40	88 (19,6%)	46 (17,0%)	24 (15,1%)	158 (18%)
40<Age<60	217 (48,2%)	151 (55,9%)	95 (59,7%)	463 (52,7%)
Age≥60	145 (32,2%)	73 (27,1%)	40 (25,2%)	258 (29,4%)
Pain duration (in yrs)				
1 yr or less	74 (16,1%)	44 (16,2%)	24 (15,0%)	142 (15,9%)
more than 1 up to 5 yrs	220 (47,8%)	151 (55,5%)	67 (41,9%)	438 (49,1%)
more than 5 yrs	166 (36,1%)	77 (28,3%)	69 (43,1%)	312 (35%)
Pain frequency in past 7 days				
Always present	383 (83,3%)	245 (90,1%)	147 (91,9%)	775 (86,9%)
Occasionally	77 (16,7%)	27 (9,9%)	13 (8,1%)	117 (13,1%)
Type of pain				
Non neuropathic	99 (24,3%)	48 (19,4%)	43 (29,23%)	190 (23,7%)
Mixed evidence	189 (46,3%)	104 (42,1%)	65 (44,2%)	358 (44,6%)
Neuropathic	120 (29,4%)	95 (38,5%)	39 (26,5%)	254 (31,7%)
*Means ± SD*				
Average pain in past 7 days	6,52 ± 2,0	6,94 ± 1,9	7,07 ± 1,6	6,75 ± 1,9
Worst pain in past 7 days	7,92 ± 1,9	8,39 ± 1,6	8,67 ± 1,3	8,20 ± 1,7
Pain interference (BPI-10)	52,74 ± 22,0	59,61 ± 22,2	61,19 ± 19,5	56,38 ± 21,9
Physical quality of life (SF-12v2 score)	30,72 ± 9,4	28,41 ± 8,1	28,32 ± 7,8	29,58 ± 8,8
Mental health–related quality of life (SF-12v2 score)	41,52 ± 11,8	39,87 ± 11,7	38,87 ± 11,7	40,47 ± 11,8

BPI-10 = Brief Pain Inventory-10; SF-12v2 = Short-Form-12 Health Survey Version 2.

Among the users of opioids, close to two thirds (63.1%) stopped taking them during the follow-up period, mainly because of side effects (36.2%) and/or lack of efficacy (20.5%).

### GEE analyses

**Figure 2.**
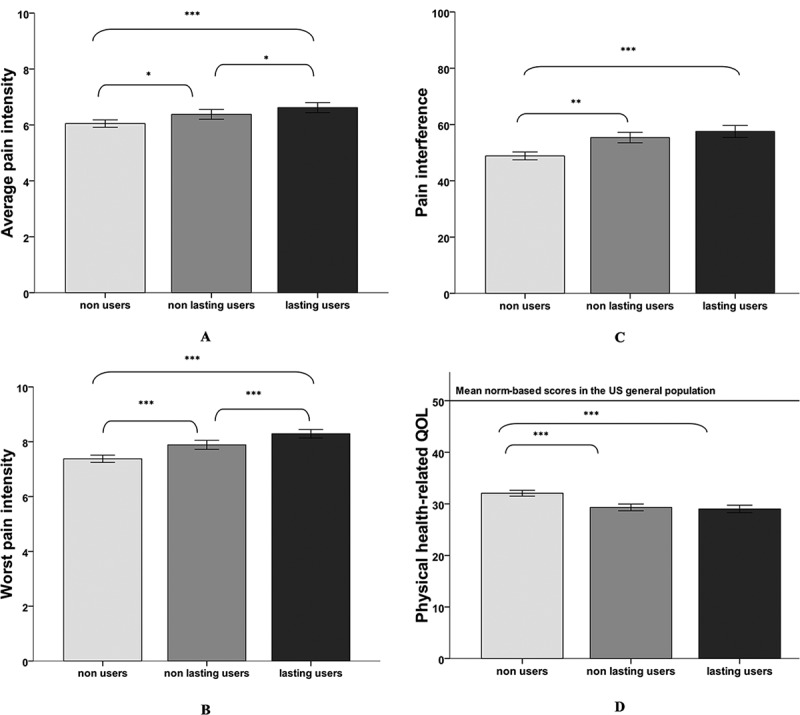
Results of the generalized estimating equations analyses. Error bars: 95% confidence interval. *P < 0.05. **P < 0.01. ***P < 0.001.

Results of the GEE, which are summarized in Table 2, showed that after adjusting for age, sex, pain duration, and frequency, the patient’s OUP was a statistically significant predictor of average pain intensity, χ^2^ (df = 2) = 20.41, *P* < 0.001, worst pain intensity, χ^2^ (df = 2) = 54.13, *P* < 0.001, pain interference, χ^2^ (df = 2) = 20.89, *P* < 0.001, and pQOL, χ^2^ (df = 2) = 28.29, *P* < 0.001, over time (OUP × time, all *P *> 0.05). This was true irrespective of the type of pain the patients were suffering from (neuropathic, nonneuropathic, or mixed evidence of neuropathic pain; OUP × type of pain, all *P* > 0.05). As shown in Figure 2, pairwise comparisons revealed that lasting users reported higher pain intensity (average pain, *P* < 0.001, Cohen’s *d *= 0.27, and worst pain, *P* < 0.001, Cohen’s *d *= 0.46), greater pain interference (*P* < 0.001; Cohen’s *d *= 0.38), as well as poorer pQOL (*P* < 0.001, Cohen’s *d* = 0.35) over time than the nonusers. Compared to nonusers, non-lasting users reported higher pain intensity (average pain, *P* < 0.05, Cohen’s *d *= 0.14, and worst pain, *P* = 0.001, Cohen’s *d *= 0.23), greater pain interference (*P* < 0.05; Cohen’s *d *= 0.26), and poorer pQOL (*P* < 0.001; Cohen’s *d *= 0.30). Comparison between non-lasting users and lasting users revealed that the latter group reported higher pain intensity (average pain, *P* < 0.05, Cohen’s *d *= 0.11, and worst pain, *P* < 0.001; Cohen’s *d *= 0.21) during the follow-up period. All of the above Cohen’s *d* values were less than ±0.5, thereby questioning the clinical significance of the observed differences.

**Table 2.** Results of the generalized estimating equation analyses.

Predictive variable	χ^2^	df	*P* value
Average pain intensity			
Opioid use profile	20.408	2	<0.001
Type of pain	6.981	2	0.05
Time	84.732	1	<0.001
Sex	0.316	1	0.574
Age	7.402	1	0.007
Pain duration	28.957	2	<0.001
Pain frequency	26.073	1	<0.001
Worst pain intensity			
Opioid use profile	54.127	2	<0.001
Type of pain	15.384	2	<0.001
Time	88.432	1	<0.001
Sex	0.877	1	0.349
Age	0.305	1	0.581
Pain duration	37.005	2	<0.001
Pain frequency	19.775	1	<0.001
Pain interference			
Opioid use profile	20.890	2	<0.001
Type of pain	8.479	2	0.14
Time	67.182	1	<0.001
Sex	1.082	1	0.298
Age	1.646	1	0.199
Pain duration	42.196	2	<0.001
Pain frequency	38.336	1	<0.001
Physical quality of life			
Opioid use profile	28.287	2	<0.001
Type of pain	1.955	2	0.376
Time	43.747	1	<0.001
Sex	0.536	1	0.464
Age	15.432	1	<0.001
Pain duration	6.032	2	<0.05
Pain frequency	17.027	1	<0.001
Mental health–related quality of life			
Opioid use profile	5.153	2	0.076
Type of pain	4.548	2	0.103
Time	17.672	1	<0.001
Sex	0.680	1	0.410
Age	14.813	1	<0.001
Pain duration	9.414	2	<0.05
Pain frequency	9.285	1	<0.005

Examination of the types of response among the lasting users of opioids revealed that at 12-month follow-up, close to one in four lasting users experienced a significant improvement (≥20%) in their pain intensity and interference. The same was true for their mQOL. In contrast, pQOL improved from baseline to 12 months among only 8% of the lasting users (see Table 3).

**Table 3.** Types of responses among lasting users of opioids over time (12 months).

Outcome	Change^a^	% of patients
	Improved	27.7
Average pain intensity	Remained stable	61.1
	Deteriorated	11.2
	Improved	21.6
Worst pain intensity	Remained stable	72.4
	Deteriorated	6
	Improved	25
Pain interference	Remained stable	68.1
	Deteriorated	6.9
	Improved	7.9
Physical quality of life	Remained stable	85.1
	Deteriorated	7
	Improved	20.2
Mental quality of life	Remained stable	69.3
	Deteriorated	10.5

^a^A change of at least 20% (for pain intensity and interference scores) or at least one SD (for quality of life scores) was considered meaningful (improvement or deterioration). Patients with a change below 20% (or below one SD for quality of life scores) were considered stable.

## Discussion

To our knowledge, this multicenter study is the first to have examined the effectiveness of long-term opioid therapy for reducing pain severity and improving health-related QOL in a large heterogeneous sample of patients with CNCP newly initiated on this type of treatment and followed in a real-life context. Our study revealed statistically significant differences in the patients’ outcomes according to their OUP. However, in studies involving large sample sizes like the present one, examination of effect sizes may prove to be more informative than statistical significance testing.^42^ In this article, none of the group differences reached a medium to large size effect (all Cohen’s *d* < 0.5). Although Cohen’s criteria are guides rather than absolutes, the clinical significance of the mean group differences can be viewed as questionable. In other words, although a significant subgroup among those who took opioids over a 1-year period did experience improvement in pain severity (intensity and interference) and mQOL, the majority did not and continued to report high pain severity scores as well as poor health-related quality of life (mental and physical).

### Use of opioids for CNCP

Results of our study showed that nearly half of patients referred to specialized pain clinics and who were not discharged before the end of the 1-year follow-up period were on opioids at one time or another during the course of their treatment, a finding that is consistent with Moulin et al.’s results.^25^ Studies carried out in primary care settings showed much lower prevalence rates of opioid use ranging from 7.1% to 19.9%.^21,46–48^ The high rate of opioid use among our sample may be explained by the fact that these patients were recruited in tertiary care centers and have typically tried many other alternative therapies without significant success.^49^ However, as important, if not more important, to emphasize is the high percentage of patients (63.1%) who interrupted the use of opioids during the follow-up period, because this can be viewed as an important treatment failure rate. These results are consistent with those of the literature reviews of Noble et al.,^50^ Furlan et al.,^20^ and Abdel Shaheed et al.,^51^ who reported high rates of opioid discontinuation across studies mainly due to adverse events and/or insufficient pain relief.

### Opioid effectiveness for reducing pain severity

The lasting opioid users continued to report high mean pain intensity scores (average and worst pain) during the follow-up period. This result is consistent with those of two studies that found that long-term opioid therapy did not contribute to lower pain intensity on average among patients with CNCP.^21,22^ In one of these studies, persistent opioid use was defined based on dispensed opioid volume and number of prescriptions during 365 days,^22^ whereas in the other, patients were considered long-term opioid therapy users if they reported using this type of medication on a regular basis.^21^

Our study also showed the same pattern of results with regard to the effects of long-term opioid use on pain interference in various aspects of daily living. In his systematic review, Ballantyne reported that function has been investigated in a limited number of trials, and these studies vary considerably in both their design and principal findings.^52^ Since then, several other studies have assessed functional outcomes among patients with CNCP using long-term opioid therapy and found that opioids did not improve physical functioning and disability.^23–26^

Relatively little scientific evidence exists on the reasons why long-term opioid treatment may fail to improve patients’ pain severity. It is commonly believed that sustained administration of this type of medication can be accompanied by a tolerance phenomenon leading to a progressive loss of the drug effects with a decrease in the apparent analgesic efficacy.^4^ Another possible reason for the lack of long-term opioid effectiveness may be related to the fact that this type of medication can also induce hyperalgesia, a phenomenon called “opioid-induced hyperalgesia” (OIH) and defined as a state of nociceptive sensitization caused by exposure to opioids and characterized by a paradoxical response whereby a patient receiving opioids for the treatment of pain may actually become more sensitive to certain painful stimuli.^53–55^ However, the precise mechanism of OIH is not yet understood, and this phenomenon has been mainly studied in the context of a short-time exposure rather than a long-time exposure to opioids.^54^ That patients with CNCP on long-term opioid therapy may develop OIH cannot be excluded, and this issue certainly merits further investigation.

### Opioid effectiveness for improving health-related quality of life

In our sample, lasting users of opioids continued to report poor QOL on the average. These results are consistent with Eriksen et al.’s^21^ and Campbell et al.’s^23^ observations suggesting that long-term use of opioids would not be really helpful for improving health-related quality of life. This is problematic in view of the fact that the amelioration of patients’ quality of life commonly constitutes the primary goal of CNCP management, a goal that is often set as important as reducing the pain itself.^56–58^ In an earlier study, Choinière et al found that health-related quality of life was remarkably low in patients with CNCP on waitlists of multidisciplinary pain treatment.^59^ Similar results were obtained in the present study, where the patients’ norm-based scores on the physical scale of the SF-12v2 were much lower than those in the general population, and this was true whether or not they were on opioid treatment. These findings further highlight the deteriorated quality of life of this group of patients.

### Response to treatment among the lasting users of opioids

Results from this study showed that though the majority of lasting users remained stable over time, about one in five patients experienced a significant improvement in their pain condition and mental health–related quality of life. This is similar to the findings of Moulin el al.’s study where close to one fifth of patients with neuropathic pain treated with opioids in tertiary care pain centers showed clinically significant improvement in their pain and function 12 months later.^25^ This is not negligible, because these results suggest that long-term use of opioids may be effective for a subgroup of patients with CNCP. Further research is clearly needed to identify the characteristics of patients who are most likely (and least likely) to benefit from long-term opioid treatment. Some earlier reports^60–63^ suggest that factors such as sex, depression, anxiety, and treatment expectations may play a role, but additional studies involving large sample sizes are needed to address this important issue.

### Study limitations

Like any other studies, the present one has limitations that merit comments. First, our sample did not include 476 patients who were discharged from the pain clinic within the 12-month follow-up and therefore did completed all follow-up questionnaires. Thus, we may have missed some patients who continued to use opioids and were doing well or patients who did not respond to opioids and were discharged from the clinic because no other treatments could be offered. Second, in our sample, the follow-up period was limited to 12 months, though it may take more than a year to really be able to assess the effectiveness of long-term opioid therapy. Third, this study is limited to patients with CNCP attending tertiary care pain clinics. Most of them had chronic pain for years and had tried several approaches to treating their condition. In addition, this study does not capture patients who are doing well on opioids in the community and who are not referred to pain clinics. This may impact the generalizability of the findings. However, one can suspect that opioids are more commonly prescribed at the tertiary care level because many family physicians may not feel comfortable using this type of medication due to the risk of substance abuse.^64^ Furthermore, it is important to mention that access to tertiary care clinics in the province of Quebec requires a physician referral; access to these clinics is free but limited due to relatively long waiting lists, as is the case in other Canadian provinces.^65^ As a result, it is unclear how the data obtained in the present study compare to what would be obtained in other health care systems (self-referrals or other systems of access to the specialized pain clinics). In addition, patients were classified into different OUP categories based on their self-reported current medication use at the time of the first visit at the pain clinic and at follow-up interviews at 6 and 12 months; it is possible that patients interrupted their opioid treatment for a while within each 6-month period separating the follow-up interviews, and this was not taken into account in the OUP classification. Finally, because our study was not a randomized controlled trial, it is unknown whether or not persistent opioid users’ conditions would have worsened over time if they had not taken this type of medication.

### Conclusions

Close to one quarter of patients who were lasting users of opioids experienced a significant improvement in terms of pain intensity, pain interference, and mental health–related quality of life. The majority of opioid-naïve patients who were followed at the pain clinic for a 1-year period did not benefit from opioid therapy, and a great proportion of them discontinued opioids due to lack of effect or presence of side effects. Results are in line with the literature suggesting that though most patients do not benefit from long-term opioid therapy, there is a significant subgroup of patients who do benefit from this therapy. The challenge facing clinicians is how to identify who the responders will be.
